# Machine learning-assisted mid-infrared spectrochemical fibrillar collagen imaging in clinical tissues

**DOI:** 10.1117/1.JBO.29.9.093511

**Published:** 2024-09-27

**Authors:** Wihan Adi, Bryan E. Rubio Perez, Yuming Liu, Sydney Runkle, Kevin W. Eliceiri, Filiz Yesilkoy

**Affiliations:** a University of Wisconsin-Madison, Department of Biomedical Engineering, Madison, Wisconsin, United States; b University of Wisconsin-Madison, Department of Electrical and Computer Engineering, Madison, Wisconsin, United States; c University of Wisconsin-Madison, Center for Quantitative Cell Imaging, Madison, Wisconsin, United States; d University of Wisconsin-Madison, Department of Computer Science, Madison, Wisconsin, United States; e Morgridge Institute for Research, Madison, Wisconsin, United States

**Keywords:** mid-infrared spectral imaging, machine learning, second harmonic generation, fibrillar collagen imaging, tumor microenvironment, cancer

## Abstract

**Significance:**

Label-free multimodal imaging methods that can provide complementary structural and chemical information from the same sample are critical for comprehensive tissue analyses. These methods are specifically needed to study the complex tumor-microenvironment where fibrillar collagen’s architectural changes are associated with cancer progression. To address this need, we present a multimodal computational imaging method where mid-infrared spectral imaging (MIRSI) is employed with second harmonic generation (SHG) microscopy to identify fibrillar collagen in biological tissues.

**Aim:**

To demonstrate a multimodal approach where a morphology-specific contrast mechanism guides an MIRSI method to detect fibrillar collagen based on its chemical signatures.

**Approach:**

We trained a supervised machine learning (ML) model using SHG images as ground truth collagen labels to classify fibrillar collagen in biological tissues based on their mid-infrared hyperspectral images. Five human pancreatic tissue samples (sizes are in the order of millimeters) were imaged by both MIRSI and SHG microscopes. In total, 2.8 million MIRSI spectra were used to train a random forest (RF) model. The other 68 million spectra were used to validate the collagen images generated by the RF-MIRSI model in terms of collagen segmentation, orientation, and alignment.

**Results:**

Compared with the SHG ground truth, the generated RF-MIRSI collagen images achieved a high average boundary F-score (0.8 at 4-pixel thresholds) in the collagen distribution, high correlation (Pearson’s R 0.82) in the collagen orientation, and similarly high correlation (Pearson’s R 0.66) in the collagen alignment.

**Conclusions:**

We showed the potential of ML-aided label-free mid-infrared hyperspectral imaging for collagen fiber and tumor microenvironment analysis in tumor pathology samples.

## 1 Introduction

The tumor microenvironment (TME) is compositionally and structurally heterogeneous, hosting a complex network of biomolecules that encode a variety of biological signals, and metabolic and immune interactions. Collagen is the dominant structural protein in the extracellular matrix (ECM) of the TME.^1^ Among the 28 types of collagen, fibrillar type I collagen forms a triple-helix structure and organizes itself into a fiber-like structure,^2^ being the primary collagen in the ECM. It has been shown that the changes in the collagen (especially type I collagen) structure or distribution are linked to many diseases including cancer. Specifically, parameters such as fiber density and orientation of collagen fibers have a significant impact on the progression and treatment of cancer.^3^^–^^6^ For example, the stroma in the TME of pancreatic ductal adenocarcinoma (PDAC) is highly fibrotic, constituting up to 85% of the tumor volume.^7^ PDAC fibrosis affects the efficacy of cytotoxic therapies and can compromise drug delivery.^2^ Therefore, comprehensive analysis of fibrillar collagen in the TME is critical for understanding the role of collagen’s architectural changes in carcinogenesis and metastasis, which have implications for the development of effective cancer therapies and personalized treatments.

Conventional histological staining agents, such as Masson’s Trichrome, Movat’s Pentachrome, Van Gieson’s stain, and Picrosirius Red, can be used for collagen imaging.^2^^,^^8^ While these labeled techniques enable inspection of collagen in tissue samples using standard widefield microscopes, they are fairly laborious and time-consuming, requiring specialized protocols to overcome stain variation effects. Moreover, histological staining-based techniques are limited in resolving collagen fibers with high resolution and providing quantifiable metrics needed for prognosis and treatment studies. The implementation of artificial intelligence models to enhance the diagnostic potential of collagen-stained images has shown promise, but it is limited by the lack of insight into the biochemistry and the detailed morphology of the ECM.^9^^,^^10^

Label-free collagen-specific imaging modalities, such as polarized light^11^^–^^13^ and second harmonic generation (SHG) microscopy, have been demonstrated to overcome shortcomings of conventional stain-based imaging. Specifically, SHG is a second-order nonlinear coherent scattering process that is highly specific to non-centrosymmetric fibrillar collagen and its supramolecular fiber morphology. In addition, SHG microscopy has sub-micrometer resolution and can perform optical sectioning of tissue with an imaging depth of up to hundreds of micrometers.^14^^,^^15^ SHG has been used to study tumor-associated collagen signatures^3^^,^^16^ (TACS); therefore, it is suitable to be ground truth in our study. In particular, TACS-3, a pattern exhibiting high fiber alignment perpendicular to tumor boundaries, has been shown to be a negative prognostic factor in breast cancer.^17^ Moreover, similar morphological signatures have also been found in other types of cancer such as skin,^18^^,^^19^ ovarian,^4^^,^^20^ prostate,^21^ and pancreas.^6^^,^^22^ While SHG imaging has elucidated the biomedical consequences of architectural changes in TME, the molecular mechanisms that drive collagen alterations are still poorly understood. Thus, there is a need for a multimodal approach where SHG and chemical imaging methods sensitive to molecular changes are employed together in TME investigations.

Infrared absorption spectroscopy is a label-free analytical technique that provides quantitative biochemical information by probing the vibrational bands of functional biomolecules. Specifically, in the mid-infrared (MIR) fingerprint region (∼800 to $1800\;{\mathrm{cm}}^{-1}$), spectrochemical analysis of measured transmittance through a specimen reveals compositional information. Recently, tunable quantum cascade lasers (QCL) with high spectral power output enabled the development of wide-field MIR spectral imaging systems that can operate at room temperature with compact footprints.^23^^–^^25^ The QCL-based MIR spectral imaging (MIRSI) can rapidly collect hyperspectral datasets from whole-slide tissue sections and has the unique capability to combine spatial and chemical information.^26^ Previous important studies revealed that the MIR spectral analysis can detect different collagen types,^27^^,^^28^ identify fibrosis in numerous tissues, including the liver,^29^ heart,^30^ and bone marrow,^31^ and provide critical prognostic information based on reactive stroma in TME-based investigations.^32^^,^^33^ In these past reports, the MIRSI-identified fibrotic regions were primarily referenced to stained images of adjacent tissue sections. However, an objective benchmarking of MIRSI-detected collagen fibers with respect to the ground truth SHG microscopy on the same tissue sample has not been performed.

**Fig. 1:**
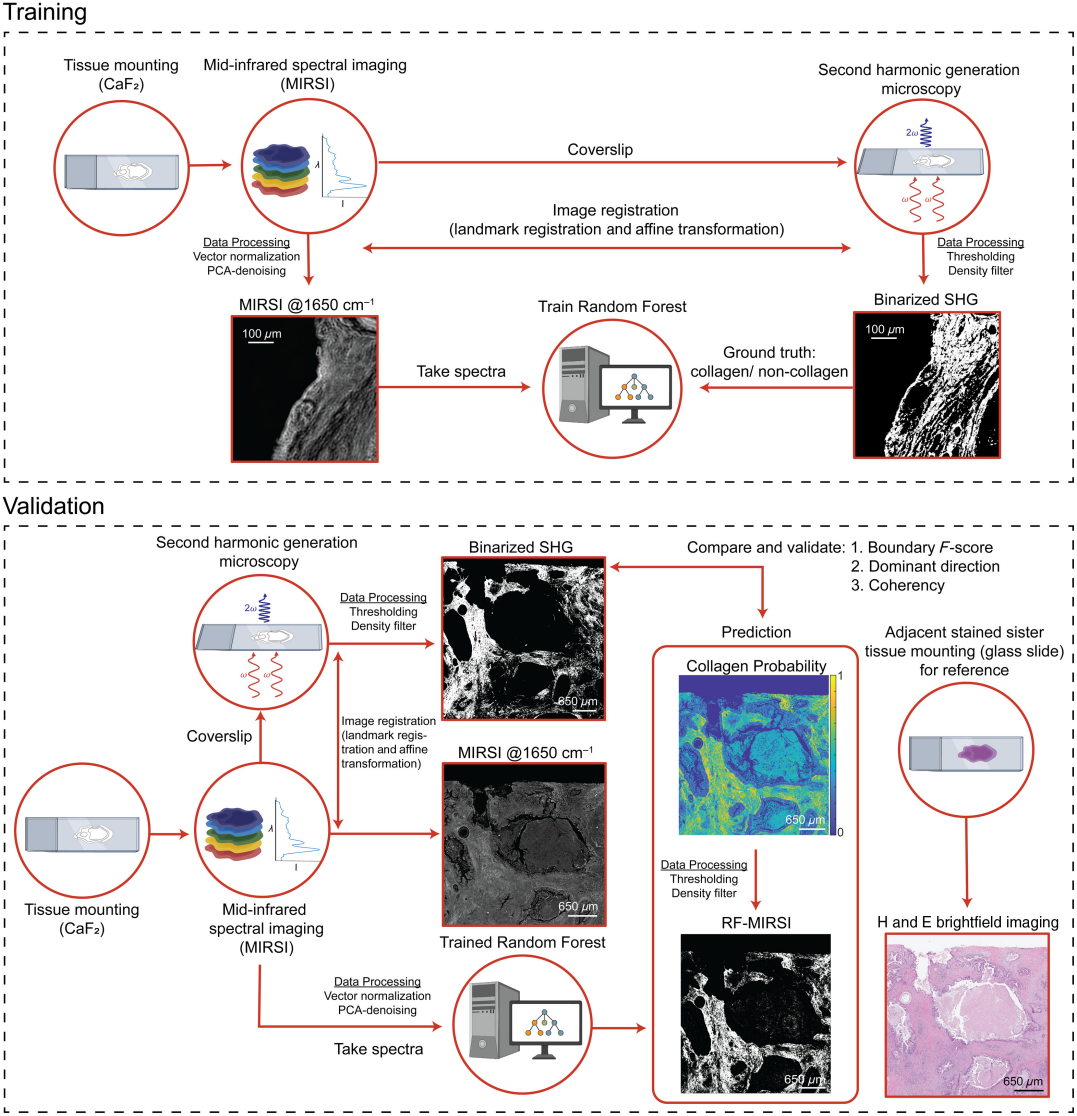
Schematic diagram of the workflow for label-free multimodal imaging using MIRSI and SHG: RF training workflow (top): a pancreatic tissue section is first mounted onto an infrared-transparent ${\mathrm{CaF}}_{2}$ substrate for MIRSI imaging. Subsequently, the tissue section is enclosed with a coverslip and imaged with a SHG microscope. Binarized SHG images are used as ground truth for the RF model training after the registration of MIRSI and SHG images. Validation workflow (bottom): once the RF model is trained, an unused independent subset of the image data is used to validate the RF-MIRSI results. To generate SHG-like images from the RF-MIRSI predicted collagen pixels, further image processing, such as thresholding and density filtering, is conducted. Finally, RF-MIRSI images are compared with SHG images that have also undergone further processing, such as thresholding and density filtering, to generate binarized SHG images for equivalent comparison. Adjacent tissue slices are stained with H&E and imaged on a standard brightfield microscope for reference. Scale bars are 100  μm for both images in the training section and 650  μm for all images in the validation section. SHG, second harmonic generation; MIRSI, mid-infrared spectral imaging; and RF, random forest.

Here, we present a new label-free multimodal imaging approach using MIRSI and SHG imaging modalities to sequentially acquire complementary chemical and morphological information from the same biological tissue sample (Fig. 1). We first developed a protocol that enabled reliable image acquisition from the same tissue section using two different microscopes, which employ two distinct frequency regions of the electromagnetic spectrum, i.e., visible-near-infrared (λ=890  nm) and MIR (λ=5 to 10  μm). To classify fibrotic regions in pancreatic tissue samples based on collagen’s spectral signatures, we trained a random forest (RF) model using large hyperspectral MIRSI datasets and SHG images of the same tissues as the ground truth. This RF model, which we named RF-MIRSI, was then used to identify regions of high collagen composition from the whole-tissue sections. Finally, we validated the RF-MIRSI fibrotic collagen segmentation method, referencing our findings to the SHG images. The RF-MIRSI method achieved a high average boundary F-score (0.8 at 4-pixel thresholds) in the collagen distribution, high correlation (Pearson’s R 0.82) in the collagen orientation, and similarly high correlation (Pearson’s R 0.66) in the collagen alignment. Our label-free multimodal collagen fiber imaging approach is a key step towards future comprehensive tumor tissue investigations where both morphometric and chemometric information are considered to better study tumor-promoting ECM alterations.

## 2 Materials and Methods

### 2.1 Tissue Sample and Pure Collagen Sample Preparation

Five human pancreas tissue samples were selected from archival, formalin-fixed paraffin-embedded (FFPE) blocks that were created at the Translational Science Biocore BioBank of the University of Wisconsin Carbone Cancer Center. Each FFPE block contains one pancreas tissue sample of one patient. The diagnosis of the patients was not retrieved since it was not needed for this proof-of-concept imaging study. The Translational Research Initiatives in Pathology Laboratory of the University of Wisconsin-Madison prepared the tissue sections for this study using Richard-Allan Scientific Cytoseal 60 Histology Mounting Media. Specifically, two adjacent 5-μm-thick sections were cut from each block and then deparaffinized. Among these two tissue sections, one was first hematoxylin and eosin (H&E) stained and then enclosed with #1.5 coverslip for later use in bright-field imaging; the other section was unstained, first put on a ${\mathrm{CaF}}_{2}$ substrate for MIRSI imaging and then #1.5 cover-slipped for the following SHG imaging. ${\mathrm{CaF}}_{2}$ was used as a substrate for MIRSI imaging because it does not have any spectral signature in our MIR spectral window. For the spectrum in Fig. S2 in the Supplementary Material, pure human type I collagen (Cat # CC050, Sigma Aldrich, St. Louis, Missouri, United States) of 1  μL volume was drop casted on ${\mathrm{CaF}}_{2}$ substrate, was dried for 2 h, and was acquired using the same MIRSI microscope described in Sec. 2.3.

### 2.2 Second Harmonic Generation Microscopy

All slides in this study were imaged with a custom-built SHG backward detection multiphoton microscope described previously.^34^ In this system, a Coherent Chameleon Ultra II Ti:Sapphire laser (Coherent, Santa Clara, California, United States) was used to deliver 890 nm light to the sample using a 20×/0.75  NA air immersion objective (Nikon, Melville, New York). The backward SHG signal was filtered with a bandpass filter (445/20  nm, Semrock, Rochester, New York) and collected with a H7422P-40 GaAsP photomultiplier tube (Hamamatsu, Hamamatsu, Japan). Circular polarization was implemented for the SHG light source. All images were collected at 512  pixels×512-pixel resolution (1  pixel=0.96  μm) with consistent acquisition settings using in-house developed acquisition software WiscScan.^35^ The images were then stitched together for each slide using the Fiji Grid/Collection Stitching plugin.^36^

### 2.3 Mid-infrared Spectral Imaging

The MIR spectral images were acquired using the Spero-QT (DRS Daylight Solutions Inc., San Diego, California, United States), which uses four tunable QCLs that span the spectral region of 950 to $1800\;{\mathrm{cm}}^{-1}$ with $2\;{\mathrm{cm}}^{-1}$ resolution. The microscope was run in transmission mode using a 12.5× air objective (0.7 NA). The linearly polarized infrared light transmitted by the tissue sample was measured at room temperature by a 480×480 microbolometer focal plane array (image pixel size: 1.35  μm). The sample chamber was kept inert with nitrogen gas. To scan an entire tissue sample, a translational stage was used to image the region of interest (ROI) consisting of multiple field of views (FOVs) as illustrated in Fig. S1 in the Supplementary Material. At the time of the measurements, the device suffered some instability at the spectral region of 1440 to $1480\;{\mathrm{cm}}^{-1}$, where the switching between two different QCLs happens. Therefore, the region was omitted from further analysis. For this work, MIRSI data from five tissue sections were collected, totaling ∼300 FOVs for validation and ∼60 FOVs for training, each with 480×480  pixels, summing up to ∼68 million spectra and ∼13.5 million spectra for validation and training, respectively. Overall, we combined RF-MIRSI FOVs encompassing all five tissues into 33 large ROIs, each of size $1950\times 1950\;\mu m^{2}$ (1440×1440 hyperspectral pixels). In each ROI, spectra from non-tissue regions (on average about 20%) were excluded.

### 2.4 Spectral Data Processing

The infrared spectral images were processed with MATLAB (MathWorks, Natick, Massachusetts, United States) using the methods described before.^26^^,^^37^ The raw hyperspectral images consist of data cubes with dimensions of 480×480×405 for each FOV. To exclude spectral data from the non-tissue regions, the protein-associated amide I absorption peak was considered because proteins are omnipresent in any tissue sample. The presence of the amide I spectral peak was determined by taking the intensity difference between $1655\;{\mathrm{cm}}^{-1}$ (amide I) and $1760\;{\mathrm{cm}}^{-1}$, and if the value lies between 0.1 and 2.0 a.u., then it implies the presence of a significant amide I infrared spectral peak and that pixel was labeled as tissue associated and considered in the following workflow. Subsequently, all the spectra excluding the spectral region of 1440 to $1480\;{\mathrm{cm}}^{-1}$ were normalized so that its Euclidean norm was set to unity. To reduce the noise in the hyperspectral MIRSI data, a principal component analysis (PCA) was first performed on the raw datasets. Only the first 40 principal components were kept (average 99.8% of variance) to reconstruct the spectra in the original wavenumber space to reduce the noise.^26^ The PCA noise reduction procedure was done separately for training and validation datasets.

### 2.5 Random Forest Model

The training and validation datasets were labeled based on the ground truth SHG images. SHG images were first registered to MIRSI hyperspectral images using landmark registrations in MATLAB as described in Ref. 38. Briefly, matching points were selected manually to indicate common points between both images using MATLAB’s Control Point Selection (cpselect) function.^39^ Then, the affine transformation that matches their scaling, translation, and rotation was determined using MATLAB’s fitgeoform2d function^40^ and applied to the SHG image using MATLAB’s imwarp function.^41^ The matched SHG images were then binarized to provide a classification label (collagen/non-collagen) for every MIRSI spectra. The SHG images were binarized by first choosing a proper background threshold. The threshold within an image was set to a level manually such that dark counts (signals from outside of tissue region) were mostly removed while the remaining true collagen signals were maximized. Across all used SHG images, the threshold was set to be ∼5% of the maximum intensity. Next, a density filter was applied to filter out isolated collagen pixels from the binarized SHG images, i.e., those with less than six neighboring collagen pixels, and henceforth is referred to as non-structural collagen. For RF model training, two MIRSI ROIs from two different tissue samples deemed representative of the whole dataset (assessed through their corresponding SHG images) were chosen and yielded ∼13.5 million spectra. There was no overlap between training and validation ROIs. From the training spectra, those that were not from tissues and surpluses were discarded to avoid class imbalance between collagen and non-collagen. In total, 1.4 million collagen and 1.4 million non-collagen labeled spectra were used for training. An overview of the training and validation data can be found in Table S1 in the Supplementary Material.

The TreeBagger class^42^ from MATLAB was used to train the RF model. The number of trees was set to 50. Increasing the number of trees to 500 did not yield any perceptible advantage; therefore, 50 was kept. As per the default recommendation, the number of features used for each decision split was set to 21 by rounding up the square root of the number of features (n=405). The accuracy of the RF model was quantified using out-of-bag error, which is equivalent to cross-validation for the RF algorithm.^43^ The model achieved an accuracy of 79% calculated using the MATLAB function oobError.^44^ Furthermore, the importance of each feature for the classification was determined by taking the feature out of the model and calculating the model performance drop based on a randomly selected training dataset (out-of-bag dataset). This calculation of the feature importance was done using the built-in implementation in MATLAB by setting the “OOBPredictorImportance” parameter to on, as detailed in Ref. 42.

### 2.6 Image Processing and RF Model Evaluation

Based on the RF model training, we first generated a new set of RF-MIRSI images of all five tissue sections, where each pixel value yields collagen probability. These probability maps, where collagen and non-collagen classes for each pixel are specified, were used in our method validation. The pixels whose probability of being collagen was higher than 50% were classified as collagen pixels and the rest as non-collagen pixels. This rather low probability threshold was chosen to accurately detect collagen-containing pixels, and a 50% threshold value was used throughout our analysis unless otherwise noted. However, this threshold resulted in many isolated pixels from non-structural collagen that can be considered noise in our study. To remove this noise from RF-MIRSI images, we applied a density filter, where only pixels with at least six surrounding predicted collagen pixels were kept, filtering out non-structural collagen. For validation, the corresponding binary images of RF-MIRSI were compared with the binarized SHG images (details on binarization were given in Sec. 2.5).

To validate the collagen distribution from the RF-MIRSI model, we calculated a metric named boundary F-score (BF-score). The F-score is the harmonic mean of the precision and recall values and is defined as $\frac{2*\text{precision}*\text{recall}}{\left(\text{recall}+\text{precision}\right)}=\frac{\mathrm{TP}}{\mathrm{TP}+\frac{1}{2}(\mathrm{FP}+\mathrm{FN})}$ with TP, FP, and FN true positive, false positive, and false negative, respectively. F-score ranges from 0 to 1, and 1 indicates perfect classification.^45^ The BF-score is an extension of the F-score that takes distance error tolerance into consideration by defining a boundary to determine whether a point matches the ground truth or not. It was calculated using MATLAB’s BF-score function^46^ for all 33 ROIs that encompass all five tissues as described in Sec. 2.4. The BF-scores were calculated with various pixel thresholds up to 8 pixels (1 pixel represents 1.3  μm, and the largest diffraction limited point is ∼10  μm). Similar calculations were done using RF-MIRSI images that were obtained at different collagen probability thresholds.

To examine the capabilities of our technique for studying collagen morphology, we calculated both dominant orientation and coherency for each selected ROI. Both metrics were obtained by calculating the structure tensor of each ROI using the Fiji plugin OrientationJ.^47^ The dominant direction is defined as the primary orientation of the ROI, ranging from 0 to 180 deg. The coherency is an indicator of fiber alignment within an ROI, ranging from 0 to 1, with “0” indicating isotropic fiber orientations and “1” indicating fibers aligned in one direction. For both metrics, the size of ROI was set to 480×480  pixels ($650\times 650\;\mu m^{2}$) to obtain the granularity of the direction. In total, 297 480×480  pixels ROIs were studied.

Pearson’s R-value was calculated using MATLAB’s corrcoef function^48^ to evaluate the correlation between the RF-MIRSI images and SHG images for both dominant orientation and coherency. Pearson’s R-value is a measure of the strength of the linear correlation between variables, ranging from −1 to 1, where −1 indicates a perfect negative linear relationship, 0 indicates no linear relationship, and 1 indicates a perfect positive linear relationship.

## 3 Results

### 3.1 RF Training and RF-MIRSI

**Fig. 2:**
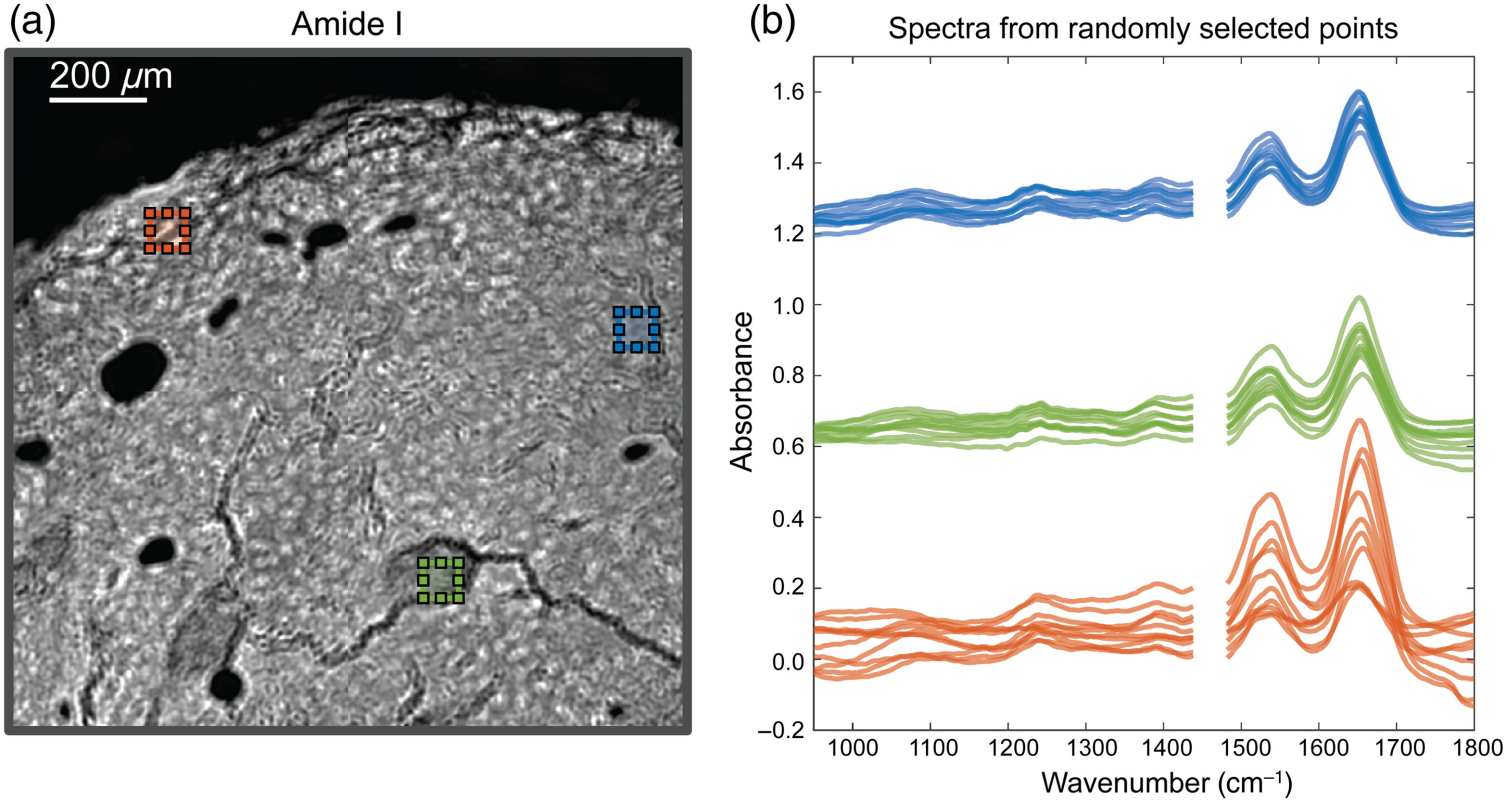
Representative MIRSI data. (a) MIR image collected at the $1650\;{\mathrm{cm}}^{-1}$ protein amide I band. (b) The MIR spectra of each of 10 randomly chosen pixels from three ROIs in a pancreatic tissue section. The data from the spectral range of 1460 to $1480\;{\mathrm{cm}}^{-1}$ was omitted due to the QCL switching issues with our instrument.

MIRSI data of five tissues were collected, constituting ∼300 FOVs of 480×480  pixels or around 68 million spectra. To illustrate our raw MIRSI spectral data, Fig. 2 shows a typical unprocessed MIRSI image collected at $1650\;{\mathrm{cm}}^{-1}$ illumination that corresponds to the protein amide I band along with 10 randomly selected spectra for each of the three outlined ROIs. The example MIRSI image consists of four (2×2) stitched FOVs collected from a pancreatic tissue section. To train the RF, 2.8 million spectra, including collagen and non-collagen pixels (1.4 million each), were used. These training spectra were pre-processed to exclude non-tissue regions and reduce noise. For the ML training, SHG image pixels were used as classification labels. MIRSI images are registered to SHG images using landmark registration, which matches their scaling, translation, and rotation. Subsequently, the trained RF model was applied to the rest of the spectra collected from the tissues. For each pixel-associated spectrum, the RF outputs the probability of how likely that pixel contains collagen. More details about the data pre-processing workflow and the training of the RF can be found in Sec. 2.

### 3.2 Correlation Between Binarized RF-MIRSI and SHG Images

**Fig. 3:**
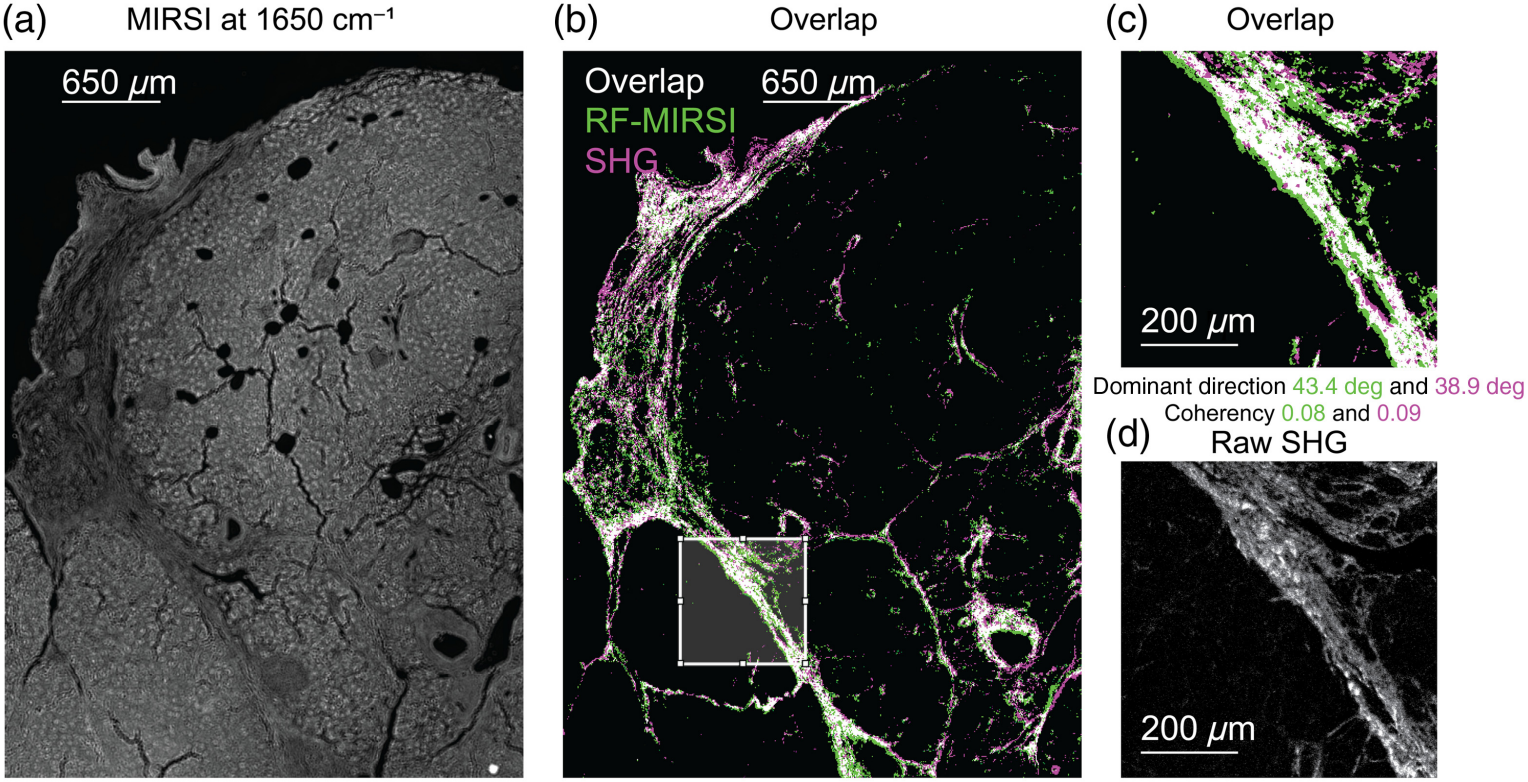
Correlation between RF-MIRSI and SHG images. (a) MIRSI image collected at $1650\;{\mathrm{cm}}^{-1}$ protein amide I band of a representative tissue. (b) Overlay of corresponding RF-MIRSI-predicted collagen image (green) and SHG image (pink), where overlapping pixels (white) indicate correspondence in between. (c) A subregion enclosed with the white box from panel (b) presenting calculated dominant direction and coherency. (d) Raw SHG image of the same region in panel (c) is shown for reference. The scale bars for panels (a) and (b) are 650  μm and 200  μm for panels (c) and (d).

Figure 3 presents the results of our correlation investigation between the RF-MIRSI-predicted collagen pixels and the SHG ground truth. A representative MIRSI image of a tissue region collected at $1650\;{\mathrm{cm}}^{-1}$ protein amide I band illumination is shown in Fig. 3(a). The RF-MIRSI predicted collagen pixels from the same tissue region are shown in green in Fig. 3(b). To qualitatively illustrate the correlation between RF-MIRSI-predicted and SHG-identified collagen pixels, Fig. 3(b) shows the binarized SHG image in pink and the pixels that are identified as fibrillar collagen by both methods in white. Collagen fiber’s dominant direction and coherency were also calculated for a randomly chosen subregion, outlined with a white box in Fig. 3(b), and the findings were presented in Fig. 3(c). For reference, an unprocessed SHG image (raw) counterpart of the same subregion is shown in Fig. 3(d). A similar analysis using the rest of the data was also performed and will be explained below.

**Fig. 4:**
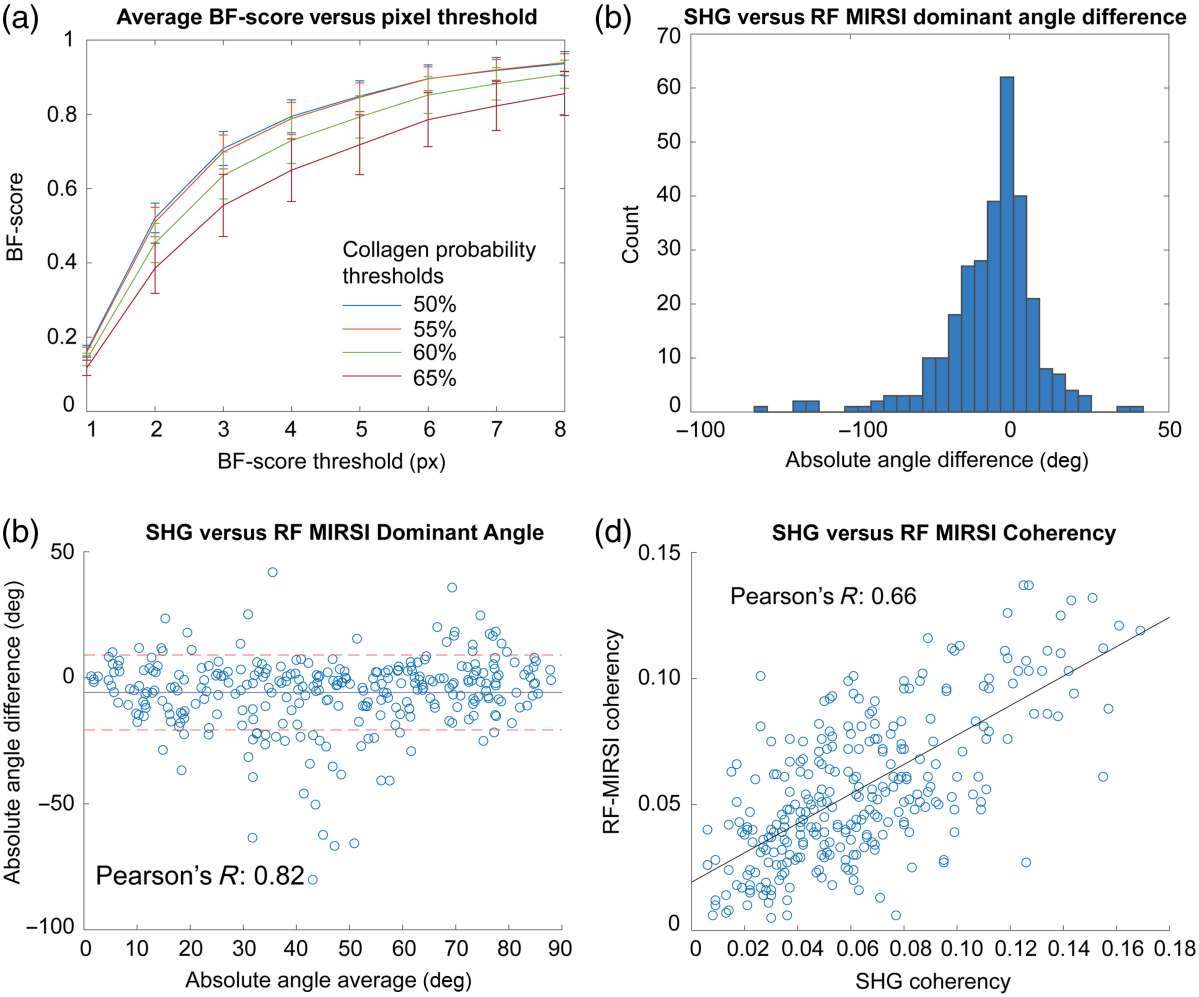
Quantitative validation of RF-MIRSI-identified collagen based on ground truth SHG images. (a) BF-score calculated to validate RF-MIRSI collagen prediction accuracy based on binarized SHG using various pixel and collagen probability thresholds. Error bar indicates standard deviation. (b) Bland-Altman plot comparing the dominant direction of collagen calculated using OrientationJ for both RF-MIRSI collagen probability and SHG images along with its Pearson’s R-value. (c) The distribution of the calculated absolute angle difference. (d) Alignment (coherency) calculation between SHG and RF-MIRSI along with its Pearson’s R-value.

For the quantitative evaluation of our multimodal imaging technique, we used BF-score, dominant angle, and coherency as metrics. We first divided RF-MIRSI data from five tissues into 33 large ROIs of size $1950\times 1950\;\mu m^{2}$ (1440×1440 hyperspectral pixels). The average BF-scores calculated for each of the 33 ROIs are shown in Fig. 4(a) for various pixels and collagen probability thresholds. For the dominant direction and coherency calculations, we used smaller ROIs (480×480  pixels) to preserve granularity. The Bland-Altman plot shown in Fig. 4(b) depicts the agreement of the dominant direction for both SHG and RF-MIRSI measurements with Pearson’s R of 0.82. Figure 4(c) depicts the distribution of the absolute angle difference between the two techniques with a mean of −5.8  deg and a standard deviation of 14.9 deg. The coherency calculated from both techniques is shown in Fig. 4(d) with Pearson’s R of 0.66.

### 3.3 Wavenumbers Significant in Detecting Collagen as Identified by Random Forest

**Fig. 5:**
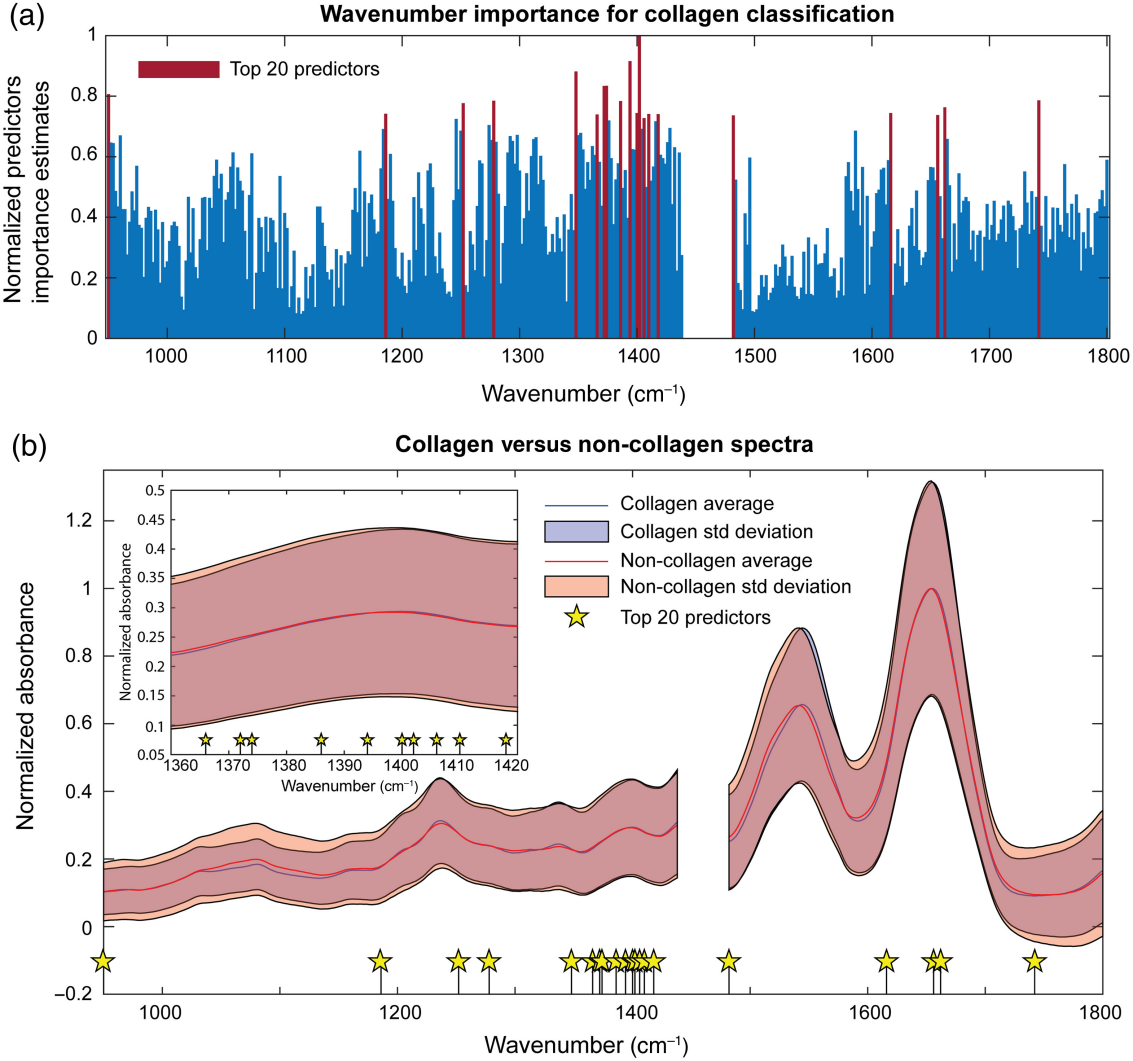
Importance of each spectral wavenumber identified by the RF model for collagen prediction. (a) The predictor importance values at each wavelength; the top 20 most important predictors are highlighted in maroon. (b) Average collagen and non-collagen spectra normalized to their respective maximum from the training dataset, together with the average of its standard deviation (shaded area).

Figure 5(a) shows the wavenumbers as a function of their importance in detecting collagen as identified by the RF model. The top 20 best predictor wavenumbers are colored in maroon. To further examine this, the average and standard deviation of collagen and non-collagen spectra from the training data are shown in Fig. 5(b) with the top 20 wavenumber predictors indicated with stars.

## 4 Discussion

In cancer, the growth of fibrous tissue around the tumor (also referred to as a desmoplastic reaction) has been shown to be an important hallmark of TME, where it presents distinct similarities to the wound healing response.^49^^–^^51^ Previously, highly organized fibrillar collagen patterns in TME were identified as negative cancer prognostic markers using SHG imaging, which is the current “gold standard” in fibrillar collagen studies.^6^ Despite their important prognostic potential, stromal-based biomarkers are not yet part of the current clinical histopathology because SHG is a low-throughput imaging technique and requires extensive expertise and costly instrumentation. MIRSI is a label-free and rapid hyperspectral digital tissue imaging technique, which can collect whole-slide tissue images within minutes. Here, we trained a machine learning (ML) algorithm using the gold-standard SHG images to generate fibrillar collagen maps using the rapid and high-throughput MIRSI modality. Our qualitative (Fig. 3) and quantitative (Fig. 4) investigations revealed that RF-MIRSI-predicted fibrotic collagen tissue regions correlate well with the gold-standard SHG imaging. Therefore, our method has the potential to contribute to the histopathology workflow by providing fibrillar collagen maps of whole-tissue pathology sections. In addition to providing fibrillar collagen maps, where there has been significant effort using other emerging technologies such as polarization microscopy^12^^,^^13^ to emulate SHG with lower cost, MIRSI takes advantage of potential biochemical signatures associated with specific diseases. Moreover, infrared photonics is a blooming field, and the costs of IR light sources and detectors are decreasing. Due to the emerging developments in the biophotonics field, the technology transfer of our proposed technique, collecting structural and chemical information from tissue samples, has a strong potential to make an impact in clinical settings.

Notably, we report a significant difference between an MIR image of a tissue section collected at a single wavelength illumination associated with the protein band [Fig. 3(a)] and the collagen map predicted by the RF-MIRSI [Fig. 3(b)]. This highlights the strength of our RF-MIRSI method in parsing out most of the tissue regions as non-collagen areas, even though they contain significant amounts of protein as evidenced in Fig. 3(a), the amide I absorption signal. Moreover, contrary to traditional single-channel spectrometry, hyperspectral imaging modalities can collect spatially and spectrally rich datasets. When evaluated using ML and artificial intelligence models, MIR hyperspectral imaging can provide versatile information that is critical in the biomedical field.

In multimodal imaging, accurate image registration is inherently challenging, especially when images are collected by different instruments with distinct spatial resolution limits, as in the case of our SHG/RF-MIRSI approach. To tackle the unavoidable image registration errors, we used the BF-score instead of the standard F-score as a validation metric. The average BF-score is calculated for all 33 ROIs encompassing all five tissues with various pixel and collagen probability thresholds as shown in Fig. 4(a). Our results show that the average BF-score reaches ∼0.8 within 4-pixel thresholds for the collagen probability threshold of 50% and 55%, which indicates a good correlation between both sets of images. Below 4-pixel thresholds, the average F-score is lower due to the misalignment in the registration of the images. The misalignment sources can include (i) human error while picking common registration landmarks for registration, which is amplified because SHG and MIRSI images are collected at different magnifications (20× versus 12.5×), and (ii) mismatching non-affine deformations that come from the different imaging system such as barrel distortion as well as the cover slipping process for the SHG imaging (sample for MIRSI imaging is uncovered). While different factors impact the multimodal image alignment accuracy, using 4-pixel thresholds (equivalent to ∼5  μm, which is close to the spatial resolution limit of MIRSI) in BF-score calculation successfully addresses this issue. Apart from misalignment, other factors that contribute to the deviations between the SHG and RF-MIRSI results can also be explained by either the inherent differences between the two processes (SHG versus absorption), such as their cross-sections and depth of field, or the difference in our implementation (SHG with circularly polarized and MIRSI with linearly polarized light), and finally, the performance of the algorithm itself.

Moreover, our quantitative validation results in Fig. 4(a) show that the average BF-score decreases with increasing collagen probability threshold. This can be explained by the lower true positive classification outcomes when a larger threshold is applied to RF-MIRSI images. Therefore, in this work, we first detected as many collagen-classified pixels as possible using a 50% collagen probability threshold, then minimized the non-structural collagen using a density filter (see Sec. 2). A similar strategy and threshold were used while binarizing the SHG images.

We also compared the morphology in the RF-MIRSI collagen maps and SHG images via the dominant angular direction parameter calculated in individual ROIs [Fig. 4(b)] and identified a high correlation (Pearson’s R of 0.82). This is also evident in Fig. 4(c), where the histogram distribution of the absolute angle differences between the imaging modalities peaks close to zero. Furthermore, the alignment (coherency) calculated for both techniques showed a high degree of correlation [Pearson’s R 0.66, Fig. 4(d)]. The outliers in Figs. 4(b)–4(d) can be mainly attributed to the following three factors: (1) the performance mismatch between the two imaging modalities, (2) the performance of the RF algorithm itself, and (3) the coverslip effect where MIRSI acquisition was done on non-coverslipped samples and SHG was on coverslipped samples. Application of the coverslip can alter the geometry in some of the regions of the tissue and can give an uncorrelated result between both modalities. Regardless, the overall results indicate that the RF-MIRSI images generated from MIRSI spectral data correlate well with the SHG images.

Among the ML models, RF has the advantage of quantifying the importance of data features in the decision-making process. It has this advantage, because it keeps the linear independence of the features, unlike many other ML models that mix multiple features, thus abstracting its physical interpretation. Moreover, RF is also a nonlinear algorithm, which is suitable for our molecular fingerprint dataset. We showcase this advantage by calculating the wavenumber importance by quantifying the increase in error generated by excluding a specific spectral feature (see Sec. 2). Figure 5(a) shows the 20 highest-ranked predictors. In MIRSI-based collagen studies, the spectral focus is usually on the protein-associated amide I and amide II bands.^52^^–^^59^ Our RF model also identified four features falling within the amide I 1600 to $1700\;{\mathrm{cm}}^{-1}$ range among the top 20 highest ranked. Interestingly, the RF model heavily relied on the spectral region between 1360 and $1420\;{\mathrm{cm}}^{-1}$, with 10 out of the top 20 predictors residing there, even though the average collagen and non-collagen spectra are not different in that region [Fig. 5(b)]. A small window (1360 to $1340\;{\mathrm{cm}}^{-1}$) within this region contains the wagging vibration of the proline side chains present in type I collagen, found in biological tissues.^56^ Therefore, the dominant dependence of the RF model on this spectral region must be due to the abundance of proline and 4-hydroxyproline in collagen triple-helix (∼22% occurrence of each in type I collagen^60^). This underscores the critical role of our holistic TME analysis via multimodal imaging, as it can provide access to biochemical information from the structurally altered tissue regions. Moreover, the vibrational fingerprints of molecules depend on many factors such as the molecules’ surrounding environment, concentration, temperature, and many more. We have shown that our tandem method selectively captures the molecular fingerprint of collagen in its relevant, native tissue environment. To demonstrate this point, the spectra of pure human type I collagen are shown in Fig. S2 in the Supplementary Material, and it is distinct from the collagen spectrum detected in the tissue with the help of SHG and RF, highlighting the strength of our approach.

In future studies, specific biochemical information such as the integrity of collagen’s triple helix structure,^52^ cross-linking collagen concentrations,^52^^,^^57^ the collagen quality associated with non-enzymatic cross-linking,^59^ and many more leveraging existing databases and literature^61^ can be investigated in the context of diseases, especially those that have been previously studied using SHG or staining methods such as various organ fibrosis^62^^,^^63^ and cancers.^64^ Such investigations can help elucidate the molecular drivers behind the morphological alterations in the TME observed in various cancer grades. Similarly, MIRSI can be used to complement SHG by analyzing interactions of collagen with other important ECM molecules such as fibronectin, which cannot be detected by SHG.^65^

Our results can be further improved by employing recently developed advanced laser scanning-based MIRSI methods or photothermal imaging,^66^^,^^67^ which can achieve a higher spatial resolution and better match SHG imaging.^68^ Moreover, metasurface-enhanced MIRSI can be used to improve the sensitivity and selectivity of the absorption spectra by benefiting from the light-matter interactions at the photonic cavities with resonances tuned to the region with a high density of important predictors.^69^

In conclusion, the proof-of-concept RF-MIRSI model was successfully used to detect fibrillar collagen based on the MIRSI spectral data from pancreatic tissue samples. This technique can be adapted to other tissue types and can complement state-of-the-art imaging modalities and analytical techniques to further investigate the complex nature of fibrillar collagen in the TME.

## Supplementary Material



## Data Availability

The code, data, and materials relevant to this work can be shared upon reasonable request.
