# Supplementary material for: Machine learning-assisted mid-infrared spectrochemical fibrillar collagen imaging in clinical tissues
Source: J Biomed Opt. 2024 Sep 27;29(9):093511. doi: 10.1117/1.JBO.29.9.093511 (PMC11448345; doi:10.1117/1.JBO.29.9.093511)
Supplement: Supplementary file 1 [file JBO_029_093511_SD001.docx]

Machine learning assisted mid-infrared spectrochemical fibrillar collagen imaging in clinical tissues

Wihan Adi^1,*^, Bryan E. Rubio Perez^2,*^, Yuming Liu^3,*^, Sydney Runkle^4^, Kevin W. Eliceiri^1,3,5^, Filiz Yesilkoy^1,#^

1. Department of Biomedical Engineering University of Wisconsin-Madison, Madison, WI, 53705, USA

2. Department of Electrical and Computer Engineering University of Wisconsin-Madison, Madison, WI, 53705, USA
3. Center for Quantitative Cell Imaging, University of Wisconsin-Madison, Madison, WI 53706, USA

4. Department of Computer Science University of Wisconsin-Madison, Madison, WI, 53705, USA
5. Morgridge Institute for Research, Madison, WI 53706, USA

*Authors contributed equally
#Corresponging author: [filiz.yesilkoy@wisc.edu](mailto:filiz.yesilkoy@wisc.edu)

Supplementary Information


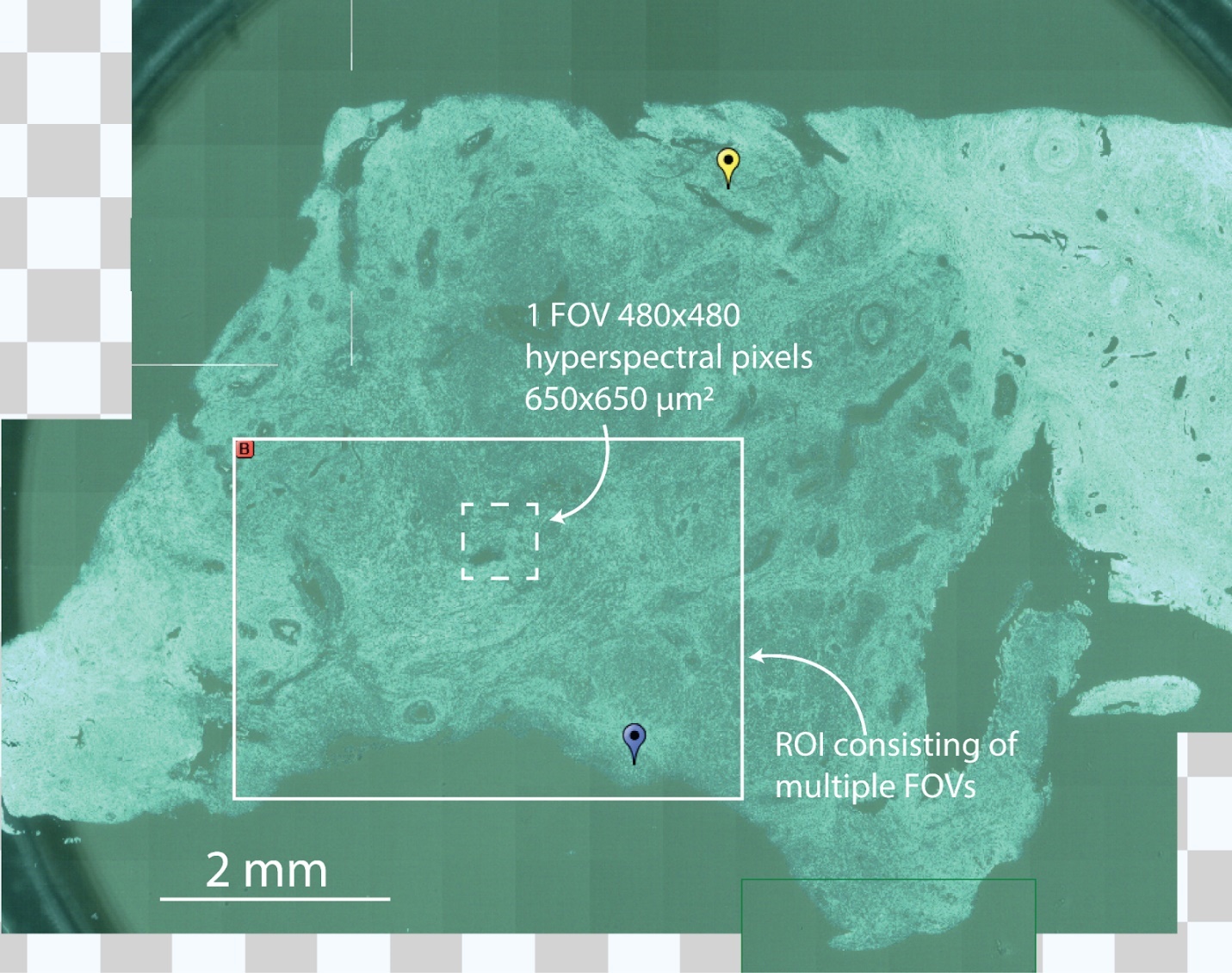

**Fig. S1.** Visible image of a tissue sample during MIRSI acquisition. MIRSI FOV (dotted square) is illustrated and acquisition of a Region of Interest (ROI) using multiple FOVs (solid square) is done using a translational stage.


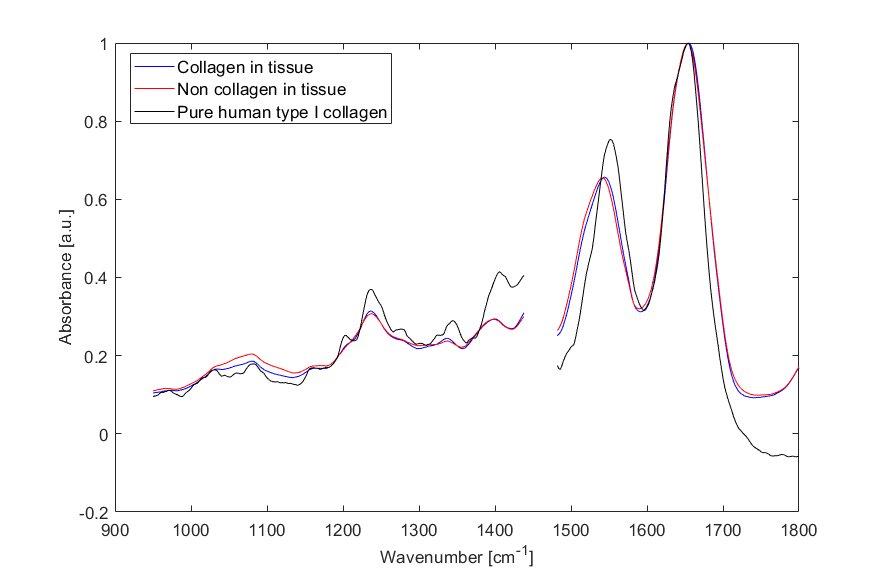


**Fig. S2.** Average spectra of pure human type I collagen (black), and average spectra of collagen- and non-collagen labeled pixels from the tissue sample as labeled by SHG (red and blue). All spectra are normalized to their respective maximum.

| Dataset | # MIRSI FOV (480x480) | # Acquired spectra | # Used spectra |
| --- | --- | --- | --- |
| Training | 59 | ~13.5 million | ~2.8 million |
| Validation | 297 | ~68 million | ~68 million |

**Table T1.** The number of acquired MIRSI FOV, acquired spectra, and used spectra for training and validation of the RF model.
